# Anthocyanins and Anthocyanin-Derived Products in Yeast-Fermented Beverages

**DOI:** 10.3390/antiox8060182

**Published:** 2019-06-18

**Authors:** Lavinia Liliana Ruta, Ileana Cornelia Farcasanu

**Affiliations:** Department of Organic Chemistry, Biochemistry and Catalysis, Faculty of Chemistry, University of Bucharest, Sos. Panduri 90-92, 050663 Bucharest, Romania; lavinia.ruta@chimie.unibuc.ro

**Keywords:** anthocyanins, pyroanthocyanins, fermented beverages, *Saccharomyces cerevisiae*, yeast

## Abstract

The beverages obtained by yeast fermentation from anthocyanin-rich natural sources (grapes, berries, brown rice, etc.) retain part of the initial pigments in the maturated drink. During the fermentation and aging processes anthocyanins undergo various chemical transformations, which include reactions with glycolytic products (especially pyruvate and acetaldehyde) or with other compounds present in the complex fermentation milieu (such as vinylphenols obtained from cinnamic acids by means of a yeast decarboxylase) yielding pigments which can be more stable than the initial anthocyanins. Overall, these compounds contribute to the organoleptic traits of the mature product, but also to the overall chemical composition which make the yeast fermented beverages important sources of dietary antioxidants. In this review, we focused on the studies regarding the changes underwent by anthocyanins during yeast-mediated fermentation, on the approaches taken to enrich the fermented beverages in anthocyanins and their derived products, and on the interrelations between yeast and anthocyanin which were of relevance for obtaining a high-quality product containing optimum amounts of anthocyanin and anthocyanin-derived products.

## 1. Introduction

**Figure 1:**
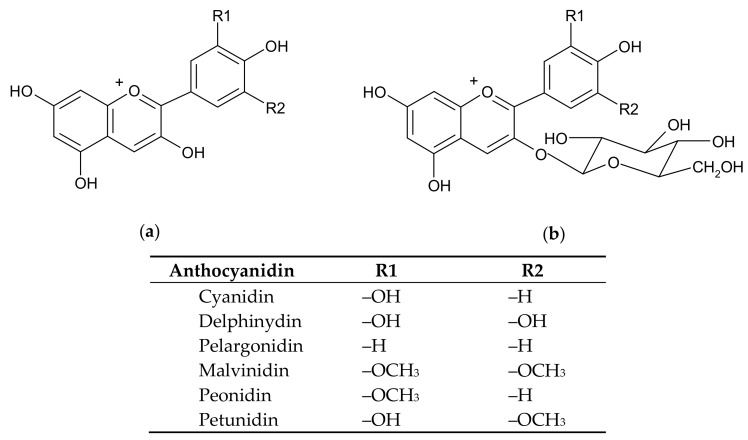
The chemical structures of the main anthocyanidins (**a**) and anthocyanins (in the form of anthocyanidin-3-*O*-glucosides) (**b**) present in plants.

Anthocyanins are water-soluble vacuolar pigments belonging to the flavonoids group which is responsible for the color of many plant organs, such as fruits, flowers, leaves, stems, tubers, and rhizomes [1]. Anthocyanins are strong antioxidants and their accumulation in plant organs is stimulated under various stress conditions, including extreme temperatures, UV irradiation, and fungal and bacterial infection [2]. Anthocyanins are glycosides (predominantly 3-*O*-β-D-glucosides) that release the aglycone anthocyanidins by means of glycosidases (again, predominantly β-glucosidases) [3]; in most cases, the aglycone moieties are more potent than the glycosylated forms with respect to the antioxidant potential [4]. The anthocyanidins are based on the flavylium (2-phenylchromenylium) ion decorated with various chemical groups which tune the spectral as well as the redox traits of anthocyanins. Although the chemical structure of anthocyanidins can vary (more than 700 anthocyanins have been described as glycosides of 27 different anthocyanidines [5,6,7]), there are six anthocyanidins which predominate in nature: cyanidin, delphinidin, malvidin, pelargonidin, peonidin, and petunidin (Figure 1), with cyanidin and delphinidin being the most abundant [7]. These compounds provide the plant organs with a plethora of red, blue or purple colors which are sensitive to variations of pH, temperature, light, oxygen, metal ions, intramolecular association or intermolecular association with other compounds (co-pigments, sugars, proteins, degradation products) [8]. The antioxidant properties of the anthocyanins relate to the high number of hydroxyl groups in the anthocyanidin moiety, the *o*-dihydroxy structure in the phenyl ring conferring higher stability to the radical forms which are encountered during electron delocalization [9].

The anthocyanins gained increasing attention due to their beneficial effect on human health. Although not essential for life, numerous studies on anthocyanins indicated that they have positive effects on alleviating the symptoms related to atherosclerosis, chronic venous insufficiency, hyperlipidemia [10], age-related macular degeneration and other eye-related diseases [11]. Also, dietary anthocyanins are considered potential regulators of obesity-derived inflammation and its associated chronic diseases [12]. It is believed that anthocyanins can even reduce body weight and insulin resistance, leading to restored glucose tolerance [13,14]. Notably, many of the health benefits brought by anthocyanin consumption are related to their potent antioxidant properties conferred by the anthocyanidin moiety [15,16,17]. With so many beneficial effects, consumption of fruits and drinks rich in anthocyanins is highly recommended on a daily basis [18].

Food sources rich in anthocyanins include all type of berries (blueberry, bilberry, blackcurrant, strawberry, wolfberry, etc.), grapes, red and purple vegetables, along with their processed products, such as beverages (wines, juices). Especially, red wines obtained from anthocyanin-rich feedstocks are considered to be an important source of dietary antioxidant when ingested with moderation. In wine research, anthocyanins are particularly important due to their effect on wine quality, playing key roles in the wine color and mouthfeel properties, as well as in wine ageing potential and stability.

In this mini-review, we included studies revealing the prevalence of anthocyanins in red wines or other fermented beverages obtained from anthocyanin-rich natural sources, with a focus on the reports showing the influence of the fermentative microorganisms—mainly yeasts—on beverage anthocyanin content and transformation. 

## 2. Yeast Fermentation Products and Anthocyanins

Red wine is one of the most widespread beverages whose health benefits are often correlated with the anthocyanin content [4,5,6,7]. Anthocyanins and their derivatives are responsible not only for the many benefits of the red wine, but also for the color of red wine, as they are transferred from grapes skin through maceration/fermentation [19]. The stability of the color is affected by wine ageing and can be enhanced through the co-pigmentation processes during vinification [20]. During fermentation, but also during wine maturation and ageing, the concentration of monomeric anthocyanins changes constantly [21]. 

**Figure 2:**
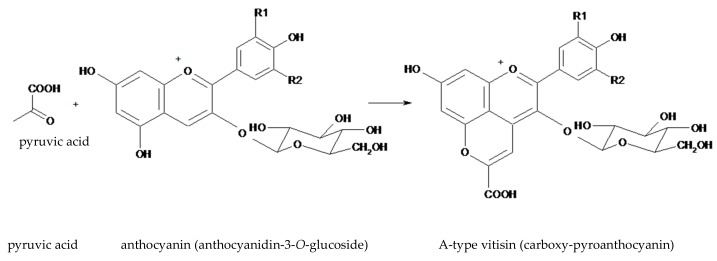
The structure of an A-type visitin (anthocyanidin-3-*O*-glucoside-pyruvate) generated from anthocyanidin-3-*O*-glucoside and pyruvic acid.

Wine making implies the yeast-mediated fermentation of simple sugars present in the must, with the final stages being dominated by the alcohol-tolerant strains of *Saccharomyces cerevisiae* [22]. In yeast, during the glycolytic fermentation of sugars, pyruvate is metabolized into acetaldehyde, which serves as a terminal electron acceptor in the generation of ethanol. The pyruvate and acetaldehyde, which are formed in yeast cytoplasm, are rapidly metabolized (pyruvate is either decarboxylated to acetaldehyde or used in the formation of acetyl CoA; acetaldehyde is reduced to ethanol) but some of these molecules diffuse out of the cell [22]. These are reactive enough to attack other molecules, facilitating the transformation of anthocyanins into a variety of compounds: pyranoanthocyanins and their secondary-generated pigments, anthocyanin oligomers, and polymeric anthocyanin pigments [20,21,22,23,24]. Among these, pyroanthocyanins are considered the most important group of anthocyanins derivatives present in fermented beverages [24]. For example, the A-type vitisins or carboxy-pyroanthocyanins (Figure 2) are produced by condensation of an anthocyanin with pyruvic acid. 

**Figure 3:**
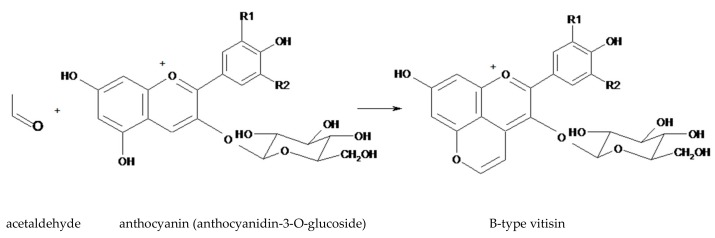
The structure of a B-type visitin (anthocyanidin-3-*O*-glucoside-4 vinyl) generated from anthocyanidin-3-*O*-glucoside and acetaldehyde.

Another group of pyranoanthocyanins are the B-type vitisins (Figure 3), which differ from A-type vitisins by lacking the carboxy group. The simplest of B-type vitisins are produced from condensation between an anthocyanin and acetaldehyde (Figure 3) [24]. 

**Figure 4:**
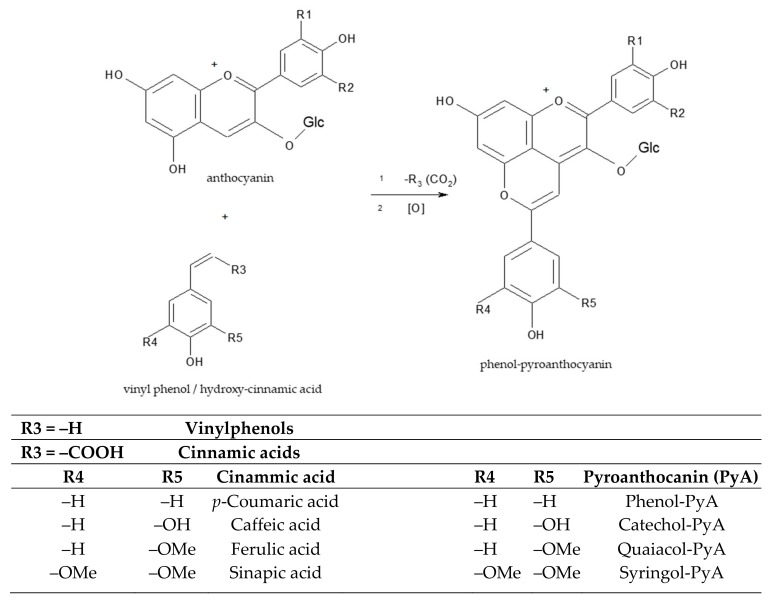
The structure of phenol-pyroanthocyanins generated from the reaction between an anthocyanin and a vinyl phenol/carboxyvinyl-phenols (*p*-hydroxy-cinnamic acids) formed in wines during fermentation [24].

Apart from pyruvate and acetaldehyde, free anthocyanins also react directly with one of the by-products of yeast fermentation, such as vinylphenols or carboxy-vinyl phenols (cinnamic acids) (Figure 4). The products have two heteroaromatic rings that show a dynamic equilibrium among different flavylium cation forms, comparatively with their reactants, the free anthocyanins [24]. 

The pyranoanthocyanins are highly stable and resistant to SO_2_ bleaching and oxidative degradation, therefore they can significantly contribute to the color stability of red wines [25], due to the formation of stable quinonoid bases which reduce the formation of non-colored carbinol bases [26]. Pyranoanthocyanins have not been detected in grape must [27], but they are found in significant quantities after fermentation, in the middle or in the final stages of fermentation via various condensation reactions. For this reason, they are poorly adsorbed by yeast cell walls because they form when the cell walls are already saturated with anthocyanins [27].

Pyroanthocyanins have common spectral characteristics with absorption maxima of 495–520 nm that are lower than those of grape anthocyanins, contributing to the red–orange color of wines developed during ageing [28]. Vinylphenol-pyranoanthocyanins especially are of great interest since they show high color stability and a bathochromic shift in their maximum absorbance towards wavelengths close to 540 nm, corresponding to bluish red color [29].

Other pyranoanthocyanins may be obtained by reaction of anthocyanins with *p*-hydroxy-cinnamic acids present in wine (e.g., caffeic, coumaric, ferulic, and sinapic acids) [24]. A colorless intermediate is first formed, but the electronic reorganization that occurs following its oxidation leads to the final molecule’s recovery of aromaticity and color [30]. This reaction is apparently slow and may occur progressively in wines over long ageing periods as well as in wines made from grape varieties with high initial caffeic acid concentrations. The reaction is favored by the presence of a high amount of *p*-hydroxy-cinnamic acids. In the presence of hydroxy-cinammate decarboxylase, hydroxycinnamic acids from grapes are conversed into vinylphenols, which readily condense with other anthocyanins to form vinylphenol-pyranoanthocyanins. Hydroxycinnamic acids are found in low concentration in grapes, usually in the form of tartaric esters that are hydrolyzed during fermentation and especially during wine ageing [31]. Another family of pyranoanthocyanin pigments are products of reactions between pyroanthocyanins with flavanol monomers or dimers [32], the products being more stable against degradation than the anthocyanins [33].

During wine maturation and ageing, the concentration of monomeric anthocyanins in red wines declines constantly. This phenomenon can be explained by the reactions presented above, but also by yeast absorption, degradation and oxidation, precipitation with proteins, polysaccharides or condensed tannins.

## 3. *Saccharomyces cerevisiae* and Vinification

*Saccharomyces cerevisiae* is a budding yeast used from the ancient times for backing, brewing distilling, winemaking, and fermented beverage production (e.g., sake, palm wine) [34]. *Saccharomyces cerevisiae* lives in the natural habitats on fruits, soil, cacti, the bark of oak trees, and the existence of a multitude of strains from different sources and environments (industrial, laboratory, and wild isolates) prompted their study under wine fermentation conditions, which refer to high levels of sugar and ethanol, high acidity, low nitrogen availability, and anaerobiosis. The traits of laboratory strains include high levels of ethyl butyrate synthesis, low biomass production, high amounts of acetate, low amounts of isoamyl acetate production, and a slow fermentation process of sugars. In contrast, the commercial wine strains ferment the available sugars completely and rapidly, producing high biomass and little acetate. No matter the type, there is a great intragroup variability for all the phenotypic traits, the wine fermentation phenotypes reflecting a wide diversity in yeast response to the environmental stress factors and adaptation to needs of specific metabolic traits [35]. 

Acidity, color, sugars and organic acids, anthocyanin content, and ethanol production are factors that generally characterize the wines. Also, there is a huge number of factors that determine the color evolution and stability of red wines, including grape variety as a sole source of anthocyanins [36]; additional methods of vinification, such as tannin addition [37] or cold maceration [38]; the selection of fermentative yeast strains that promote the formation of stable pigments through the production of metabolites, such as pyruvic acid or acetaldehyde [39], or that promote changes in pigment composition through the adsorption of anthocyanins through the yeast cell walls [38] and co-pigmentation phenomena [40]. 

To produce high-quality wines with reproducible and predictive properties, the wine distilleries use starter yeasts which produce in a short time a wine with desirable organoleptic characteristics, in terms of chemical composition, aroma, flavor, and color; this approach is often preferred because the inoculated yeast strain is predominant, being able to suppress the indigenous flora. Nowadays, there are over 200 commercial *S. cerevisiae* winemaking strains available [41] which have the ability to sustain complete fermentation of substrates rich in sugars, but poor in proteins, also producing valuable secondary metabolites [42] that interact with the must in different ways.

There is a common practice for winemakers to use selected yeasts to stabilize the red color of wines. For example, vitisins play an important role in maintaining the color of wines, especially when aged in barrels or when used to undergo a second fermentation (e.g., sparkling wines) since they are proportionally more stable than other pigments. Additionally, the color of vitisins is less affected by changes in the pH [43] or sulfur content, and they are more stable against oxidation, protecting the wine from the brown tones produced by oxidation. In practice, wine producers make the selection of the *S. cerevisiae* strains so that they produce and excrete increased amounts of pyruvate and acetaldehyde, resulting in higher concentrations of type-A and type-B vitisins (Figure 2 and Figure 3). In young wines, the anthocyanins turn into less colored forms or become polymerized with other flavonoids to produce pigments with maximum absorption of around 500 nm. Vitisins absorb at shorter wavelengths, such that the wines maintain their red tones [44].

From the dynamic point of view, during *S. cerevisiae* fermentation, type-A vitisins are formed more rapidly than type-B vitisins during the initial stages, reaching levels close to those found at the end of fermentation. This is consistent with the release of pyruvic acid, which is produced and excreted by the yeast in the early stages of fermentation when the medium is rich in nutrients. Towards the end of fermentation, when nutrients are limited, the yeast cells begin to use some of the excreted pyruvate [43]. Therefore, the highest amount of type-A vitisins is produced in the first six days of fermentation. At the end of fermentation, the amount of acetaldehyde is high, accounting for the concentrations of type-B vitisins present at the end of fermentation [27]. 

The formation of vitisins is influenced by SO_2_, pH, and temperature. It is common practice to use SO_2_ in winemaking due to its antioxidant and bacteriostatic proprieties, being added before the fermentation/maturation process. Dissolved SO_2_ is a powerful nucleophile and is capable of bonding covalently with electrophiles such as pyruvate and acetaldehyde to form adducts, preventing them to react with anthocyanins to form vitisins, and the production of vitisins in *S. cerevisiae* is reduced when increasing SO_2_ dose [27]. Temperature also greatly affects the production of fermentation metabolites like acetaldehyde and pyruvic acid, and consequently, the formation of vitisins A and B; this is why temperature must be kept in the interval 20–30 °C during fermentation [30] and with a pH no lower than 3.7 [24,27].

Red wines obtained by *S. cerevisiae* fermentation can develop increased vinylphenolic pigments if the musts/wines are supplemented with different hydroxycinnamic acids, like caffeic, ferulic acid or *p*-coumaric acids. It was reported that *S. cerevisiae* strains with high hydroxyl-cinnamate decarboxylase activity produce more vinyl-phenols, and consequently, more phenol-pyranoanthocyanins; therefore, selection of yeasts with high hydroxyl-cinnamate decarboxylase activity could increase the content of stable pigments in red wines that are to be aged for long periods; this is important especially when grapes with no color stability or grapes cultivated in problematic climatic conditions are used [45]. 

Another way to improve the quality of red wines is the release of free anthocyanidins by means of β-glucosidases. Although it was shown that β-glucosidase activity is higher in non-*Saccharomyces* yeast strains [46] and that β-glucosidase activity of *S. cerevisiae* is weakly sensitive to the presence of glucose and low pH values [47], *S. cerevisiae* strains with active β-glucosidases have been reported [48]. 

The quality of red wine is influenced by the number of anthocyanins and their products obtained during must fermentation, when the characteristics of yeast strains are of utmost importance. Remarkable, it was found that yeast itself can be an elicitor of anthocyanin production by grapes [49]. Grapes represent a natural habitat for yeasts, but yeast extract may be used by viticultors to spray the vines in order to stimulate anthocyanin biosynthesis [49].

## 4. Wines from Other Anthocyanin Containing Feedstock 

### 4.1. Fruit Wine

Fruit wines are fermented beverages which are produced using the same fermentation methods as classic wines. The anthocyanin-rich fermented fruit beverages are made from various types of ripe berries, with higher sugar content [50,51,52,53]. For example, pomegranate wines have an attractive color and antioxidant capacity due to the high content of anthocyanins in pomegranate juice [52,53], which are preserved when its fermentation is performed with *S. cerevisiae* var. bayanus, a cryophilic yeast which is active at temperatures below 22 °C [53]. Cherry wine is an important segment of the fruit industry in China, which can be processed towards high number of anthocyanins. Cherry wine also has a special aroma given by a higher content of volatile aldehydes and terpenes [54]. 

### 4.2. Rice Wine

The pigmented rice is a major component of the Asian diet. One of the important characteristics of black rice is the high phytochemical content of flavonoids, phenolics, tannin sterols, tocols, γ-oryzanols, amino acids, and essential oils [55,56]. The color of the grains is due the especially high content of anthocyanins. The fermented black rice has been studied from the point of view of the influence of fermentation conditions on anthocyanin content, the antioxidant activity, and β-glycosidase activity during fermentation by *S. cerevisiae* and efficient hydrolysis of cyanidin-3-*O*-glucoside and peonidin-3-*O*-glucoside to cyanidin and peonidin, respectively, were observed, which enhanced significantly the bioactivity of fermented black rice [57].

### 4.3. Purple Sweet Potato Wine

The purple sweet potato (*Ipomoea batatas*) is a special type of sweet potato which contains a high amount of anthocyanin pigments in its tuberous roots, being suitable for the production of health-promoting alcoholic fermented beverages with good aroma and color [58]. It was shown that anthocyanins from purple sweet potatoes are more stable than those from other plants [59]. Because *S. cerevisiae* cannot metabolize starch, exogenous glucoamylases are needed. A high starch-assimilating sake strain of *S. cerevisiae* was constructed for the production of anthocyanin-rich alcoholic beverages using starch from purple sweet potato flour by expressing *Debaryomyces occidentalis* glucoamylase gene (*GAM1*) in sake yeast, which was engineered to achieve direct liquefaction, saccharification, and fermentation for a one-step conversion of starch from purple sweet potatoes. The approach was shown to be suitable for sake, beer, and wine yeasts, yielding anthocyanin-rich alcoholic beverages [60].

## 5. Other Yeasts Used in Fermentation of Anthocyanin-Rich Feedstock

Recently, the scientific and biotechnology communities have become increasingly interested in non-*Saccharomyces* yeast species selected to solve modern challenges in winemaking. These yeasts usually perform the first stages of natural spontaneous fermentation and play important roles in wine variety. The non-*Saccharomyces* yeasts include *Kloeckera apiculata*, *Hanseniaspora uvarum*, *Hanseniaspora vineae*, *Candida zemplinina*, *Candida pulcherrima*, *Candida stellate*, *Schizosaccharomyces pombe*, *Hansenula anomaly*, *Metschnikowia pulcherrima*, *Lachancea thermotolerans*, *Kazachstania aerobia* [61,62,63,64,65], but the best studied, commercialized, and utilized at the industrial level is *Torulaspora delbrueckii* [66].

The big disadvantage of the most non-*Saccharomyces* yeasts is the fact that they have low-to-moderate alcoholic fermentation abilities, and thus cannot accomplish a proper regular fermentation process in high-alcohol beverages such as wine. This is the main reason why the most non-*Saccharomyces* yeasts must be used in combination with a more powerful fermenter, such as *S. cerevisiae*, usually in sequential fermentation to eliminate the possible inhibitory effects of the *Saccharomyces* species in the early stages of alcoholic fermentation [64]. Some non-*Saccharomyces* strains investigated in this direction are listed in Table 1. For example, strains of selected non-*Saccharomyces* wine yeasts belonging to the genera *Metschnikowia* and *Hanseniaspora* were evaluated for their effect on red wine qualities and even though they provided a higher content of acetaldehyde comparatively with *S. cerevisiae*, the concentration of B-type vitisins was lower; nevertheless, in the case of co-fermentation with *S. cerevisiae*, the combination of yeasts provided an increase in the content of B-type vitisins, probably due to the enhanced acetaldehyde formation [67].

*Schizosaccharomyces* genus include non-*Saccharomyces* yeasts that produce high amounts of undesirable metabolites such as acetic acid and hydrogen sulfide, besides pyruvic acid or acetaldehyde. The use of *Schizosaccharomyces* yeasts in the fermentation process introduce some advantages, such as lower malic or lactic acid content or high phenol-pyroanthocyanin content [68,69,70,71]. As the fermentation capacity of *Schizosaccharomyces* yeasts is lower than that of *S. cerevisiae* strains, mixed and sequential cultures with *Saccharomyces* have been used to reduce the negative effects [70]. 

Using the pre-fermentative cold maceration in the vinification processes leads to the production of high molecular weight pigments which enhance color stability. The low temperatures modify the competition between *Saccharomyces* and non-*Saccharomyces* yeasts. Two different *Metschnikowia* spp. strains (*M. pulcherrima* MP 346 or *M. fructicola* MF 98-3) and a commercial pectinolytic enzyme preparation during pre-fermentative cold maceration, followed by sequential inoculation with *S. cerevisiae* on the *Vitis vinifera* L. Sangiovese grapes (known for their problems with color stability caused by a high content of unstable and oxidizable phenols) showed a decrease of the monomeric pigments and an increase of polymeric forms, due to the bleaching process with SO_2_ [72,73]. 

Only five commercial *T. delbrueckii* strains are available to winemakers on industrial markets. Using a sequential fermentation with *T. delbrueckii*, an increase in total anthocyanins [74] was observed, but also a lower production of stable color forms, such as B-type vitisins. This effect is explained by the fact that *T. delbrueckii* is a low acetaldehyde producer [74]. Other experiments performed by sequential fermentations revealed an increase of the content of type-A vitisins, comparatively with the control *S. cerevisiae* [75]. Because the production of type-A vitisins depends on pyruvic acid [75], this fact is related to the more highly developed glycerol-pyruvic pathway reported for this species [74]. The presence of vinylphenolic pyranoanthocyanins at a few milligrams per liter in fermentations involving *T. delbrueckii* [76] but also a slight decrease in *p*-coumaric acid [77] suggested a possible hydroxycinnamic decarboxylase activity for one of the *T. delbrueckii* strains related to the formation of vinylphenolic pyranoanthocyanins [78]. Decreases in phenolic compounds such as flavanols and phenolic acids were also recorded in *T. delbrueckii* [77], which is good for selecting appropriate strains similar to *S. cerevisiae* or *Pichia guillermondii* for ageing purposes in red wine production [78]. Another *Pichia* species, namely, *P. gummiguttae* was used to obtain wines from Jamun fruits (*Syzygium cumini* (Jamun), an evergreen tropical tree) and different amounts of anthocyanins (monomeric or polymeric) were detected, depending on the presence of Jamun seeds [79].

## 6. Engineering Yeast Cells for Anthocyanin Synthesis

During yeast fermentation, some of the anthocyanins naturally released are absorbed by the cell wall of yeast and the content of anthocyanins from wine is less when the yeast is removed. From the organoleptic point of view, the anthocyanin adsorption by cell walls influences the color of wine, resulting in reduced color intensity. The cell wall of *S. cerevisiae* is highly hydrophilic and porous, and consequently, a good adsorbent for wine pigments [80,81,82]. To hamper anthocyanin adsorption during fermentation, yeast strains with low adsorption capacity can be selected [82]. To improve color stability, producing higher amounts of metabolic precursors of pyranoanthocyanins is another promising approach [83].

Perhaps one of the most straightforward ways to increase the content of anthocyanins in wines would be the utilization in the fermentation process of yeast strains engineered to synthesize de novo such compounds. Taken into account the positive effects of antioxidants on the human health, the food industry focuses constantly on producing beverages with a high content of anthocyanins. The food industry needs huge amounts of anthocyanins and the natural sources are sometimes insufficient [84]. Although numerous progress in engineering plants have been made in breeding plants enriched in anthocyanins, the process is still limited by the slow growth of plants, environmental and regional factors affecting overall yields, and difficult separation from structurally similar compounds during purification [85]. In contrast, well-studied microorganisms like *Escherichia coli*, *Saccharomyces cerevisiae*, *Pseudomonas putida*, *Corynebacterium glutamicum* are already used to large-scale production of different compounds in the food, chemical, and pharmaceutical industries, as they have numerous advantages over plants such as short time of reproduction and low costs, also providing eco-friendly synthesis alternatives, with no heavy metals, organic solvents, toxic wastes, strong acids or bases. The main advantage of using microorganisms as hosts in order to produce chemical compounds is the large availability of extensive molecular tools for their genetic manipulation, allowing heterologous expression of whole biosynthetic pathways, according to production conditions [85]. The anthocyanin synthesis is a very well understood and characterized pathway in plant secondary metabolism. Introducing the genes involved in anthocyanin biosynthesis isolated from plants may turn yeast into cell factories capable of producing anthocyanins on demand. In two studies set six years apart, *S. cerevisiae* cells were engineered to produce anthocyanins from glucose by introducing the biosynthetic genes from *Arabidopsis thaliana* and *Gerbera hybrida* in yeast genome [86,87]. Although the effective production of anthocyanins by yeast cell factories is complicated by the many secondary products obtained, the anthocyanidin synthase being identified as a major obstacle to efficient production [88], the proof of concept that *S. cerevisiae* is capable of de novo production of anthocyanins opens new perspectives for targeted anthocyanin production (Table 1).

## 7. Conclusions

In the age of functional and molecular foods, monitoring the exact quantity and type of substances which define or individualize a product is of the utmost importance. Beverages obtained from anthocyanin-rich natural sources are expected to bring many of the health and nutrition benefits which are brought by anthocyanin consumption. In this mini-review, we included studies which revealed the fate of anthocyanin during yeast fermentation of anthocyanin-rich feedstock, with a focus on the possibility to stabilize the anthocyanins during fermentation or to enrich the beverages with anthocyanins-derived products. Most of the studies done so far indicated that anthocyanins present in the must obtained from anthocyanin-rich sources undergo various transformations, both physical (e.g., adsorption by fermentative microorganisms) and chemical (out of which condensation reactions involving glycolytic intermediates such as pyruvate and acetaldehyde are most representative), greatly contributing to the organoleptic properties of wines and to color stabilization during wine ageing. Remarkable, as result of condensation with various components present in the fermentation milieu, the anthocyanin-derived products retain their initial electron-rich moieties, along with the spectral (color) and antioxidant traits. Nevertheless, while the chemical transformation of anthocyanins during yeast-mediated fermentation is well understood, less efforts have been made to unravel the antioxidant traits of anthocyanin-derived products. Although the effects of anthocyanin-rich fermented beverages or of extracts obtained from such dinks (especially red wine) on human health have been investigated [89,90,91], no comparative studies on the health effects induced by individual anthocyanin-derived products (e.g., pyroanthocyanins) obtained during yeast fermentations have been done. It is known for instance that pyroanthocyanins are chemically more stable than the parent anthocyanins, but whether the former are also biologically more active than the parent compounds is still a question to be addressed in the future.

## Figures and Tables

**Table 1:** Non-*Saccharomyces* strains used in the production of anthocyanin-rich beverages.

Yeast Tes	Anthocyanins Analyzed	Reference
*Torulaspora delbrueckii*	Vitisin A	[74,76]
*Hanseniaspora guillermondii,* *Hanseniaspora opuntiae,* *Hanseniaspora vineae, Metschnikowia pulcherrima,* *Torulaspora delbrueckii*	Vitisin B	[67,74,83]
*Torulaspora delbrueckii* *Schizosaccharomyces pombe* *Pichia guillermondii*	Vinylphenolic pyranoanthocyanins	[71,76,78,82]
*Metschnikowia pulcherrima* *Metschnikowia fructicola* *Torulaspora delbrueckii* *Pichia gummiguttae*	Total content	[72,73,74,79,88]
